# Vaginal Repair of Cystocele with Anterior Wall Mesh via Transobturator Route: Efficacy and Complications with Up to 3-Year Followup

**DOI:** 10.1155/2009/743831

**Published:** 2009-08-24

**Authors:** Robert D. Moore, John R. Miklos

**Affiliations:** Atlanta Urogynecology Associates, Northside Hospital, Atlanta, GA 30067, USA

## Abstract

*Study Objective*. The objective of this study was to report on the safety and efficacy of cystocele repair with anterior wall mesh placed via a transobturator route (Perigee system, AMS, Minnetonka, MN). *Design*. Single center retrospective study. *Setting*. Single center hospital setting and Urogynecology practice in the United States. *Patients*. 77 women presenting with symptomatic anterior wall prolapse. *Intervention*. Repair of cystocele with an anterior wall Type I soft-polypropylene mesh placed via a transobturator approach. Concomitant procedures in other compartment were also completed as indicated. *Measurements and Main Results*. 77 women underwent the Perigee procedure at our institution over a 2-year period. The mesh was attached to the pelvic sidewalls at the level of the bladder neck and near the ischial spine apically with needles passed through the groins and obturator space. Mean follow-up was 18.2 months (range 3–36 months). Objective cure rate was 93%. Subjectively only two patients have had recurrent symptoms of prolapse, and only 1 of these has required repeat surgery for cystocele. Mesh exposure vaginally occurred in 5 patients (6.5%); however all were treated with estrogen and/or local excision of exposed mesh and had no further sequelae. There were no incidences of chronic pain, infection, or abscess, and no patient required complete mesh removal for infection, pain, or extrusion. *Conclusion*. In select patients with anterior wall prolapse, repair with mesh augmentation via the transobturator route is a safe and effective procedure with up to 3 years of follow-up.

## 1. Introduction

Pelvic organ prolapse (POP) is a significant health issue in females worldwide [1, 2]. There are approximately 250 000 procedures annually in the US for POP, with many women having to undergo repeat surgery for failure of previous procedures [2]. Traditional anterior repair of cystocele or anterior wall prolapse utilizing the patient's own tissue is a compensatory procedure that has reported high failure rates and can result in vaginal shortening and/or constriction. Additionally, plication or colporrhaphy techniques address only midline defects and attaches poor-quality tissue to poor quality tissue, under tension, which most likely contributes to the high failure rate associated with these types of repairs. Paravaginal repair completed abdominally or laparoscopically is a more anatomic procedure; however it still relies on reattaching the patient's own native tissue, that has already failed, back out to the arcus tendineus (white line), and has never been proven to be more effective in long-term cure rates than vaginal anterior repair [3]. 

Graft use in prolapse surgery is somewhat controversial, however it has been proven to be very effective and has become a standard of care in the treatment of severe apical prolapse. Abdominal Y-mesh sacralcolpopexy has the highest cure rates in literature for vault prolapsed, and the benefit of utilizing mesh in the repair seems to outweigh the risks [4]. It results in anatomic repair with minimal tension and does not rely on the patients weakened tissue to maintain support. With the success of apical graft use, more recently, graft augmentation of prolapse repair has been utilized via the vaginal route. Julian first reported on the use of synthetic mesh for cystocele repair in 1996 [5], and more recently there have been multiple reports of various methods to place grafts via a vaginal approach for cystocele repair [6–19]. Although improved cure rates compared to traditional repairs have been reported, most of these techniques require advanced surgical skills, require large and difficult dissections, and can carry higher morbidity compared to traditional repairs. Many different techniques have also been reported on various means to attach the graft in place, as this has been proven to be somewhat difficult in the anterior compartment. This lack of standardization as well as the more complex nature of these repairs has resulted in slow acceptance of these techniques to utilize a graft in cystocele repair.

The transobturator space has been shown to be a very safe space for the placement of tension-free tape slings for the treatment of stress urinary incontinence and has simplified the technique of this procedure [20–22]. The space was then utilized to secure the anterior arms of a tension-free graft for cystocele repair; however no apical attachment of the graft was described [15]. The purpose of the current study is to report on the outcomes of a group of patients that underwent a standardized minimally invasive vaginal technique utilizing the transobturator space to pass needles through for assistance in placement and attachment of an anterior wall mesh for cystocele repair. The Perigee System (American Medical Systems, Minnetonka, MN, USA) is a kit that contains 4 side-specific, patented, helical needles designed for each anatomic pass through the obturator space to attach adjustable a graft to the pelvic sidewall in 4 locations. The kit was designed in attempts to help make the placement of an anterior wall graft less invasive, more simplified, and standardized. The kit includes a soft, monofilament, macroporous polypropylene mesh that has 4 self-fixating, adjustable arms with special connectors to attach to the needles that are passed through the obturator space.

## 2. Materials and Methods

This study is a descriptive retrospective case series of 77 consecutive women with symptomatic Stage 2 or greater anterior wall prolapse (cystocele) that underwent anterior repair with mesh graft augmentation with needles passed through the transobturator space utilizing the Perigee procedure over a 2-year period at our center. Comprehensive preoperative urogynecologic exams were completed including prolapse quantification utilizing the International Continence Society Pelvic Organ Prolapse Quantification (POP-Q) staging system. Additional testing included complex urodynamic testing to evaluate for the presence of concomitant stress urinary incontinence (or detrusor instability) with and without the patient's prolapse reduced. If SUI was documented on urodynamic testing, with or without the prolapse reduced, the patient was scheduled for a tension-free sling procedure at the time of surgery. A sling was not placed prophylactically in any patient if SUI was not seen on testing. All procedures were performed by the authors. 

### 2.1. Surgical Procedure

After signing informed consent, the patients were taken to the operating room and prepped and draped in the dorsal lithotomy position using adjustable Allen stirrups. Typically, the anterior compartment was addressed first if other repairs or incontinence procedures were completed. A weighted speculum and/or a self-retaining retractor was utilized to obtain exposure. The anterior wall of the vagina was infiltrated subcutaneously with a solution of 1/4% lidocaine and 1 : 400 000 epinephrine to help with the dissection plane and reduce bleeding. An anterior wall incision was made vertically in the midline starting at the level of the bladder neck and extended down the anterior wall toward the cervix or vaginal cuff, if hysterectomy was previously completed. If the uterus had no abnormalities, a hysterectomy was not completed and the uterus was left insitu. The incision was stopped 2-3 cm short of the cervix or the vaginal cuff. If hysterectomy was completed at the time of the surgery, the cuff was closed horizontally and then the anterior wall incision was made; however we made sure not to connect the two incisions; that is, a tissue bridge was left between the two incisions.

**Figure 1:**
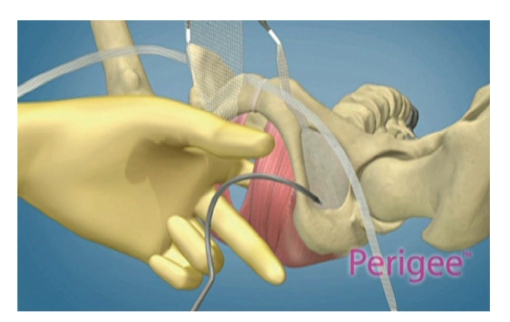
Inferior or “apical” needle being passed through the obturator space to attach the more apical arm of the mesh graft to the pelvic sidewall at the level of the ischial spine.

The vaginal epithelium was then grasped and then dissected off of the bladder and out laterally to the pelvic sidewalls up to the level of the ischial spines bilaterally. Apically and in the midline, the bladder was dissected all the way up and off the cuff of the vagina or the cervix if the uterus was in place. This dissection is essentially the same as we would complete for an anterior repair. We tried to make our dissection slightly deeper than we would for standard anterior repair and leave some of the endopelvic fascia on the vaginal epithelium in order to reduce the risk of mesh complications. This dissection leaves a thicker vaginal epithelium over the mesh once closed. After the dissection was completed, 4 small stab incisions are made in the groins. The superior incisions were made in the genitofemoral crease below the adductor longus tendon at the approximate level of the clitoral hood. The inferior incisions were made 3 cm inferior and 2 cm lateral to the superior incisions bilaterally. The needles are passed through the groin incisions and the obturator space (Figure 1) and are brought with direct finger guidance through the sidewalls at the level of the bladder neck and approximately 1.5 cm distal to the ischial spine apically. The mesh arms are then attached to the needles and they are pulled back out of the groin incisions.

**Figure 2:**
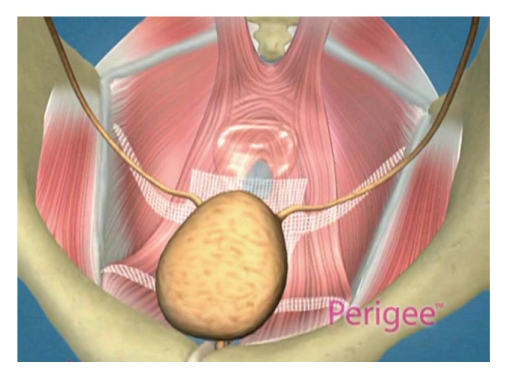
Final positioning of the mesh providing support under the bladder.

Once all the needles were removed, the tail of the mesh was cut according to the patients vaginal length and the apical aspect of the graft was attached to the pericervical ring or the cuff of the vagina with absorbable sutures. The arms were then adusted and tightened in a tension-free fashion which created a hammock-effect under the bladder and elevated the bladder back into its normal anatomic position. The final positioning placed the mesh under the bladder and attached laterally to the arcus tendineus from the bladder neck up to the ischial spine bilaterally (Figure 2). Minimal to no vaginal epithelium was excised and the incision closed with a running, locked 2-0 Vicryl suture. Prior to removing the plastic sheaths from the adjustable arms, the lateral aspects of the anterior vaginal wall were checked to ensure that none of the arms were creating any tension vaginally, and if so, a finger was used to push up in this area which would loosen the tension. The outer plastic sheats were then removed, which fixated the mesh into place. Cystoscopy was completed at the end of the case and ureteral patency confirmed with indigo carmine dye given intravenously. 

If an incontinence procedure was completed concomitantly, a separate suburethral incision was made, and the tension-free sling was placed utilizing standard technique. Remaining prolapse procedures were then completed as necessary. Postoperatively, vaginal packing and foley catheter were left in for 24 hours. If stable, patients were discharged home on postoperative day one and antibiotics were given for 5 days postoperatively. Vaginal estrogen cream was started one week postoperatively and used every other day. 

Patients were evaluated in the office at 4 weeks, 3 months, 6 months, and then every 6 months thereafter. ICS POP-Q staging was completed as well as subjective assessment of prolapse (feeling or seeing a bulge), incontinence and urinary urgency, and frequency symptoms. Objective cure was defined if the midline anterior vaginal wall (point Ba) was < or = to −1.0 cm inside the hymenal ring.

## 3. Results

Patient demographics are presented in Table 1. Of the 77 patients, 24 (31.2%) had Stage 2 prolapse, 38 (49.3%) Stage 3, and 15 (19.5%) had Stage 4 on preoperative pelvic examination and POP-Q scoring. The mean Ba value (±SD) was +2.27 ± 2.0 cm outside the vaginal opening. Thirty-one (40.2%) patients had previous anterior vaginal wall repairs and had recurrent cystoceles. Stress urinary incontinence was present in 44% of patients and tension-free tape slings were placed at the time of their surgeries. Twenty-two patients (28.5%) presented with SUI and 12 (15.5%) were discovered to have occult stress leakage with their prolapse reduced during urodynamic testing. Concomitant procedures at time of surgery included 32 patients (41.5%) with posterior repair (12 with mesh grafts, 3 with porcine dermal grafts), 29 patients (37.6%) with vaginal vault suspension (21 laparoscopic sacralcolpopexy, 8 Apogee procedure), and 2 patients with concomitant hysterectomy (1 LAVH, 1 TVH; see Table 2). Twenty-one patients (27.2%) were sexually active prior to surgery.

Average blood loss was 77cc (range 10–400cc). There were 2 intraoperative bleeds that formed hematomas under the anterior wall after initial closure that required opening up the anterior wall incision after finishing other repairs (patients were still in the operating room). The hematomas were evacuated and bleeding was controlled with sutures. One of these patients required a postop blood transfusion of 2 units of PRBCs on postoperative day 1. This was the only patient in the series that required transfusion. There were no postoperative bleeds or hematomas and no patient had to be taken back to the O.R. for bleeding or pain. There was one midline cystotomy, above the trigone, that occurred during the dissection of the anterior wall. This was closed in a 2-layer fashion with absorbable sutures and the mesh was still placed. There were no bladder injuries passing any of the needles. The average hospital stay was 1.2 days (range 1 to 4 days). Average time to void was 2.4 days (range 1–10 days). Foley catheters were taken out on postoperative day one and voiding trial attempted. If patient did not pass the voiding trial, she was sent home with an indwelling catheter and voiding trial was reattempted on postop day 3. The patient that had the complication of cystotomy had her catheter in for 10 days and had no sequelae from the injury.

Average followup was 18.1 mos (range 3–36 mos). Objective cure rate was 93.5%, using a definition of Ba </= −1.0 as considered cured. Subjectively only two patients in the series had a symptomatic failure (97.4% subjective cure rate). One patient had a repeat repair for symptoms of pressure and the other is considering repair of the failure. Mean Ba value was −2.45 ± 0.9, point C was −7.6 ± 1.3, and TVL was 9.1 cm ± 0.7. Mean vaginal length did not change statistically from preop values (Table 3). Three patients suffered from postoperative levator myalgia that required short-term treatment with a muscle relaxant. The pain resolved in all patients in less than 2 weeks and no patient suffered from long-term pain. One patient suffered from groin pain in the region of the adductor longus tendon and periurethrally on the same side. She presented at one week postoperatively with this pain. She was treated conservatively with pain medicine, muscle relaxants, and rest, and the pain spontaneously was resolved by postop week number 4. She continues to be pain free and suffered no long term weakness or other sequelae. In this predominantly elderly postmenopausal patient group, only 21% were sexually active preoperatively, and although no significant dyspareunia was reported postoperatively, no specific conclusions could be regarding sexual dysfunction.

Fifty-three patients (68%) complained of urge leakage and urge symptoms preoperatively and 39 of these patients (73%) had resolution of these symptoms postoperatively following their surgery. Three patients (3.8%) developed denovo urge symptoms postoperatively requiring treatment with anticholinergic agents. Two patients (2.5%) have had problems with recurrent UTI's since surgery.

Seven patients suffered from SUI postop. Three (4%) had concomitant tension-free slings (2 TVT, 1 TOT) at time of surgery that failed. One of these patients had repeat TVT sling and was cured, one patient had periurethral collagen and was cured, and the 3rd patient opted for no treatment. Two patients (2.7%) early in the series suffered from preop SUI; however no sling was placed as it was thought that the Perigee may be able to be used to treat SUI as well. This was found not be successful as both patients suffered from persistent SUI postop. One underwent TVT sling at 2 months postop and was cured, and the other opted for no treatment as she had very mild SUI. The last two patients (2.7%) did not have SUI preop neither subjectively nor objectively with their prolapse reduced; however they developed it postop. One had subsequent TVT sling and was cured, and the other opted for no treatment as she had very mild symptoms.

There were no postoperative infections of the mesh and no mesh had to be removed secondary to infection or pain. No patients had to be taken back to the operating room for revision of the mesh or release of the lateral mesh arms due to pain. Five patients (6.4%) suffered from mesh extrusion due to healing defects (4 discovered <12 weeks postoperatively, one at 13 months). All of the extrusions were <1 cm in size with minimal erythema or granulation tissue and no evidence of infection to the graft or tissue surrounding it. Four of the 5 patients required revision (5.1% overall revision rate) in the O.R. which was completed under local anesthesia and involved a very minor procedure of excising the exposed mesh and closing the mucosal defect. These patients all healed with no further extrusion or sequelae. The fourth patient's epithelium healed over the defect with use of estrogen cream alone and did not need revision.

## 4. Discussion

Repair of anterior wall prolapse has been one of the most challenging aspects of the pelvic reconstructive surgeon for many years and continues to be the compartment that plagues the surgeon with recurrences after repair. The search for a permanent cure of the cystocele has been going on for more than a century and continues into present day. Traditional vaginal repair of cystocele utilizing the patient's own tissue is a compensatory procedure that does not restore normal anatomy and has very high failure rates. The traditional anterior colporrhaphy plicates weakened tissue together, under tension which most likely leads to its high failure rates. Richardson identified that a large percentage of cystoceles were actually caused by tears of the pubocervical fascia away from the arcus tendineus pelvi and this caused rotational descent of the anterior wall leading to cystocele [23]. Paravaginal defect repairs have been described abdominally, laparoscopically, and vaginally, and although they are seemingly more anatomic, they have never been proven more effective than traditional colporrhaphy [3]. This may again be secondary to the fact that we are still suturing the patients own weakened tissue back together and are relying on this tissue to withhold the same forces that caused it to fail in the first place. 

General surgeons have been utilizing synthetic mesh in the repair of hernias for many years and have seen the benefit of increased cure compared to repair with native tissue under tension. Graft use in pelvic surgery has been reported on for many years; however it is only recently that its use seems to be more widespread. Although its use vaginally has been somewhat controversial, most seem to agree that grafts may be necessary to try to achieve more anatomic repairs with higher cure rates. The management of using a mesh graft in the anterior compartment is also supported by a recent Cochrane review that reported a higher rate of recurrent prolapse after anterior colporrhaphy than after mesh repair [24]. Abdominal sacralcolpopexy utilizing synthetic mesh has become the gold standard to treat severe vaginal vault prolapse because of its high success rate and excellent anatomic outcome. The graft is placed over the top half of the vagina (in the Y-mesh technique) and is brought up to and attached to the presacral ligament, a very strong attachment point. Multiple sutures are placed on the vagina, which distributes the tension and therefore does not rely on one or two sutures to suspend and hold on to the top of the vagina. Its high overall success rate may also lie in the fact that the mesh goes approximately 1/3 down the anterior wall and usually at least halfway down the posterior wall, therefore giving support to the top of the bladder and over the rectum which may reduce recurrences in these compartments as well. Some surgeons even question the need for cystocele repair when sacralcolpopexy is completed [25]. 

With the success of apical graft use, more recently, graft augmentation of prolapse repair has been utilized via the vaginal route. A review of the series that have utilized synthetic meshes for vaginal cystocele repair can be seen in Table 4. Cure rates in the range of 75% to 100% have been reported with followup ranging from one to 37 months. Although improved cure rates compared to traditional repairs have been reported, most of these techniques require advanced surgical skills, require large, and difficult dissections, and can carry higher morbidity compared to traditional repairs. Additionally, there is no standardization to these techniques, and their acceptance for general use has been slow.

de Tayrac et al., in an attempt to simplify the technique of graft placement and attachment, were one of the first to utilize the transobturator route for partial attachment of a mesh graft in the anterior compartment; however, they only utilized the space to attach the graft laterally at the level of the bladder neck [15]. The more apical portion of the graft was placed without attachment under the bladder, and no apical attachment to the sidewalls or sacrospinous ligament was utilized. Without apical attachment of the graft to the sidewalls, the mesh can be displaced, bunch up in the middle, and possibly lead to complications such as dyspareunia and/or failure over the long term. de Tayrac surmised that the failure rate they did observe might have been secondary to this tension-free placement of the mesh. 

In 2004, Rane began devising a technique to utilize the obturator space to attach the graft not only at the bladder neck but also more apically through the white line near the ischial spine utilizing needles passed through the obturator space. He felt that a strong four-point fixation of the mesh to the lateral pelvic sidewalls will lead to higher long-term cure rates. The Perigee procedure was developed based on these ideas and utilizes two different shaped helical needles passed through the obturator space to attach a mesh graft to the pelvic sidewall at the level of the bladder neck and higher up in the vagina near the ischial spine. Anatomic studies completed on cadavers have shown the needle passages to be a safe distance from the critical vessels and nerves in the space including the obturator and pudendal vessels and nerves. The dissection is the same that is utilized for anterior repair and does not require dissection into the retropubic space nor to the sacrospinous ligament therefore keeping the dissection simplified and minimizing risks of bleeding. The result of the procedure is an anterior wall mesh that supports the bladder from the bladder neck up to the ischial spine and from sidewall to sidewall.

The current study is one of the first reports in the US literature on a series of patients that underwent the transobturator Perigee procedure for anterior wall prolapse with followup greater than a year. We found the surgical procedure a minimally invasive, safe, and time-efficient method to place an anterior wall mesh for treatment of cystocele. We have previously attempted other techniques of graft placement in the anterior compartment and found them to be very time-consuming and difficult with extensive dissections and high risk of bleeding and abandoned them for these reasons. The Perigee procedure has the advantage of simplicity and standardization as it comes in a kit with a prefabricated graft and accompanying needles.

Our series showed excellent anatomic results and an objective cure rate of 93.5% (Ba </= −1.0) with up to 3 years of followup (mean 18 mos). Subjectively only two patients have suffered symptomatic prolapse (97.4% subjective cure rate) and no patient has had prolapse of the anterior wall outside of the introitus. These results are consistent with other series utilizing synthetic mesh for cystocele repair; however we believe the Perigee procedure to be less invasive and a more simplified technique to place an anterior wall graft. Thirty-one patients (40.1%) had previous repairs and would be considered higher risk for failure; however their cure rate was consistent with patients that had not had prior repair and there was no difference in complications seen in these patients either. In our opinion, this is a group of patients that benefit the greatest from a graft, given a previous failure using their own tissue, and the results of these patients in our series are very encouraging.

Concerning the safety of the procedure, bleeding was minimal in most cases with average blood loss at 77cc. Two patients did develop intraoperative hematomas under the anterior wall during completion of other procedures; however the bleeding was found to be secondary to the dissection and not the needle passes through the sidewall. One of these patients had a blood loss of 400cc and did require blood transfusion postoperatively; however our threshold for transfusion in her was lower secondary to her suffering from mild anemia preop as well as sarcoidosis with diminished lung capacity. She recovered well without any further sequelae. No patient had any postop bleeding nor required reoperation for bleeding. One patient suffered a midline cystotomy during the procedure; however this was during the dissection of the anterior wall and was not related to the mesh or needle passes. We repaired the cystotomy with a double layered closure, still placed the mesh, and she recovered without sequelae. 

Recently, concerns over complications with vaginal mesh and vaginal mesh kits have been raised [26]. Complications that may be related to the mesh itself such as mesh extrusion vaginally, pain (vaginal, groin, buttock, or leg), dyspareunia, infection, or fistula have been reported in literature [27, 28]. While these complications certainly are concerning and may occur, the overall rates of these complications seems very low both in the current study and in previous studies involving vaginal mesh and/or mesh kits [29–31]. Additionally, complications such as pain (vaginal, leg, buttock, etc.), infection, and/or dyspareunia are a risk of any vaginal repair whether it is a traditional repair without mesh or one with a mesh graft. These risks certainly do need to be balanced with the benefits of seemingly higher cure rates observed when a mesh graft is added to the anterior compartment repair. One also has to consider that different kits or techniques utilize different attachment points, different size and shape of needles, and different sizes and make-up of mesh, and therefore these all can contribute to complications.

There has also been concern of groin-pain following transobturator procedures; however we did not have any patients that suffered from long-term pain in the groin region. We feel that this is secondary to the needles being passed from the outside-in and being careful to stay below the adductor longus tendon with the superior pass as well as staying as medial as possible to the ishiopubic ramus during both needle passes. One patient did suffer from short-term groin pain and periurethral pain unilaterally; however this resolved spontaneously by 2 weeks postop. We also feel that it is very important to avoid any tension on the lateral mesh arms as this can also lead to not only potential groin pain but also vaginal pain or dyspareunia. We did have two patients that suffered from vaginal pain postoperatively and were found to have levator myalgia and pelvic floor spasm on exam. They were placed on a muscle relaxant, and both patients pain, resolved by the fourth week postoperatively. This type of pain may be secondary to the mesh arms traversing the levator muscles; however we have also seen this type of pain with traditional uterosacral vault suspension, which typically also resolves with time and muscle relaxants. We did not have any patient present with delayed groin or vaginal pain and saw no evidence of the mesh arms “tightening” or the mesh shrinking over time, which could potentially cause pain to develop further out from surgery.

There has also been significant concern over the thought that the use of mesh or mesh kits causes an increased risk of dyspareunia compared to traditional repair [32]; however this does not seem to be the case in literature. The overall risk of pain with intercourse with traditional repairs without mesh has been reported to be as high as 36% and therefore not an insignificant risk [33]. Recent studies have actually shown that vaginal mesh does not seem to have a negative impact on sexual function [34, 35], prospective comparative studies between mesh and traditional repairs in the anterior compartment have shown no significant difference in rates of dyspareunia [31], and one has actually shown a lower risk of dyspareunia in the mesh group [36]. Risk of dyspareunia or vaginal pain can be kept to a minimal by ensuring that the mesh lies flat in the space and is placed tension-free; that is, the mesh arms penetrating the sidewalls should not be pulled too tight or create a “band” as this can cause pain with or without intercourse. Additionally, as graft technology continues to improve, a lighter, less dense type I mesh may also help reduce these risks even more.

The complication of vaginal mesh extrusion has made many surgeons very hesitant to utilize synthetic mesh vaginally. In the current series, our overall rate of mesh extrusion was 6.4% (5/77), which is consistent with other reports in literature. All extrusions but one were seen prior to 12 weeks postoperatively. One patient healed spontaneously with vaginal estrogen treatment alone, and the other four required minor revision in the O.R. under local anesthesia and mild sedation (5.1% overall revision rate). We have found that when an extrusion occurs, it is typically a very small defect that can be treated easily with small excision and closure of the epithelium that does not result in long-term sequelae. No patient suffered from infection of the mesh or required removal of the entire graft secondary to infection or pain. We feel the low extrusion rate, and minimal morbidity is secondary to the mesh being a soft macroporous monofilament polypropylene (Type I) mesh, which seems to be the best tolerated graft material available today for vaginal surgery. We also feel that, by keeping the vaginal incision as small as possible, making a slightly deeper dissection, excising minimal vaginal epithelium, and utilizing pre- and postoperative vaginal estrogen help keep mesh extrusion rates as low as possible. Vaginal erosion of synthetic mesh is a common, but seemingly accepted, complication of abdominal sacralcolpopexy with an overall rate of 3.4%; however typically these erosions occur at the apex of the vagina [4]. We have found that these types of extrusions are much more difficult to manage compared to the small distal extrusions that may occur with vaginal mesh placements.

In conclusion, we have found that the vaginal repair of anterior wall prolapse utilizing an anterior wall mesh placed with needles passed through the transobturator space, a safe minimally invasive and effective procedure for the treatment of anterior wall prolapse in this subset of mostly postmenopausal patients. We feel that the role of mesh in vaginal repairs is in its infancy, and much study still needs to be done to determine the ideal material to be utilized and the optimal way to place and attach the graft vaginally and the proper patient to utilize it in. A limitation of any surgical trial that also has to be considered is surgeon experience, expertise, and skills with the particular procedure and anatomy. Complications in the current trial may have been kept to a minimal secondary to this variable (i.e., a higher level of expertise) and therefore the translation of the results to general ob/gyns and/or urologists must be considered. The current study is limited by its retrospective nature and medium-term followup and we do recommend further prospective studies with longer term followup prior to recommending its general use in clinical practice.

## Figures and Tables

**Table 1:** Demographics.

	*N* = 77
Age (years)	70.5
Parity	2.8
Previous hysterectomy	68.8 %
Menopausal	87.2 %
Estrogen use	27.4 %
Previous repair	40.2%
>1 Previous repair	7.8%

**Table 2:** Concomitant procedures.

Procedure	*n*	%
Hysterectomy	2	2.5
Vaginal Vault		
* *-Laparoscopic sacralcolpopexy	15	19.5
* *-Laparoscopic sacrohysteropexy	6	7.8
* *-Apogee	8	10.4
Posterior repair		
* *-No graft	17	22.1
* *-Mesh graft	12	15.6
* *-Porcine graft	3	3.9
Tension free slings	32	41.2

**Table 3:** Preoperative versus postop POP-Q measurements (mean).

	Preoperative	Postoperative	*P*-value
Mean POP-Q measurements			
* *Point Ba (cm), anterior wall	+2.3 ± 2.0	−2.5 ± 0.9	<0.5
* *Point C (cm), cervix or vaginal vault	−4.3 ± 4.0	−7.6 ± 1.3	<0.5
Total Vaginal Length (cm)	9.1 ± 1.6	9.1 ± 0.7	NS

**Table 4:** Review of literature of mesh use in cystocele repair.

Author	Year	Mesh	*n*	Followup (months)	Anatomical success rate (%)	Vaginal infection (%)	Vaginal erosion (%)
Julian [5]	1996	Marlex	12	24	100	0	8.3
Nicita [6]	1998	Marlex	44	3	93.2	0	2.3
Flood et al. [7]	1998	Marlex	142	36	94.4	3.5	2.1
Mage [8]	1999	Mersuture	46	26	100	0	2.2
Migliari et al. [9]	2000	Prolene	12	20	75	0	0
Hardiman et al. [10]	2000	GyneMesh	18	1	100	0	11.1
Adhoute et al. [11]	2004	GyneMesh	52	27	95	0	3.8
Shah et al. [12]	2004	Prolene	29	25	93.3	0	6.7
Dwyer and O’Reilly [13]	2004	Atrium	47	29	94	0	7
Milani et al. [14]	2004	Prolene	63	17	94	0	13
de Tayrac et al. [15]	2006	GyneMesh	63	37	89.1	0	9.1
de Tayrac et al. [16]	2007	Sofradim Soft poly-propylene	132	13	92.3%	0	6.3
*Hiltunen et al., [17]	2007	Low-weight polypropylene	104	12	93.3% versus 61.5% ant repair	0	17
*Sivaslioglu et al. [18]	2008	Polypropylene (Sofradim)	90	12	91% versus 72% (ant repair)	0	6.9
*Nieminen, et al. [19]	2008	Low-weight polypropylene	105	24	89% versus 59% (ant repair)	0	8.0

*denotes prospective randomized trial.
